# Vaccination, Revaccination, Variola, Etc.

**Published:** 1851-05

**Authors:** 


VACCINATION—REVACCINATION, VARIOLA, ETC.

At a recent meeting of the Royal Medical and Chirurgical Society, London, Dr. Webster reported a case of smallpox which occurred the third time in the same individual, and the last attack proved fatal. This victim to variolous infection was vaccinated at the age of three months. Six years after the vaccination, he and his brother had smallpox. Five years after, the brothers were again attacked with variola, and a third brother, who had been vaccinated, was taken with smallpox during the second visitation of the disease in the family. Twelve years after the second attack, one of the victims of that attack was seized with variola, in India, and died.

Dr. Stewart reported an interesting case of confluent smallpox, which occurred in a patient after a third vaccination.

These cases gave rise to an animated debate, and the general conclusion seemed to be about the same as the one we reached in our recent sketch of smallpox. The opinion of the members of the society, generally, was that vaccination is the safest and most reliable prophylactic against variola. Dr. Gregory endeavored to urge upon the Society his singular statistics, and a notion that after the fifteenth year of vaccination the only protection is in inoculation. This is certainly a very preposterous suggestion, when we reflect upon the number of second and third attacks of smallpox that occur in some persons. Dr. Gregory seems to have almost a mania for the resuscitation of inoculation. B.

